# Online Information on Antioxidants: Information Quality Indicators, Commercial Interests, and Ranking by Google

**DOI:** 10.3389/fpubh.2017.00090

**Published:** 2017-04-21

**Authors:** Romaan Aslam, Daniel Gibbons, Pietro Ghezzi

**Affiliations:** ^1^Brighton and Sussex Medical School, Brighton, UK

**Keywords:** Internet, information quality, Google, websites, antioxidants, supplements, vitamins, online information

## Abstract

The idea that antioxidant supplements can prevent or cure many diseases is extremely popular. To study the public understanding of antioxidants on the Web, we searched the term “antioxidants” in http://Google.com and analyzed 200 websites in terms of typology (news, commercial, professional, health portal, no-profit or government organization, scientific journals), disease or biological process mentioned (aging, immunity, neurological disease, diabetes, arthritis, etc.), and stance toward antioxidants, whether neutral, positive, or negative. Commercial and news websites were prevalent (over half of the total) but not in the top 10 returned by Google, where the most frequent were health portals, government, and professional websites. Among the diseases mentioned, cancer was the first, followed by vascular and eye diseases. A negative stance toward supplements was prevalent in the whole search, and this was even more evident for cancer. Information on aging or immunity had the largest proportion of pro-supplement and commercial websites. This study shows that some diseases are highly associated with antioxidants on the Internet and that information on antioxidants in aging and immunity is more likely to describe the positive effects of antioxidant supplements.

## Introduction

As early as 1956, Harman proposed the free radical theory of aging and suggested that chemical antioxidants could slow down the aging process (1). The idea that oxidative stress, defined as an imbalance between production of reactive oxygen species and the endogenous antioxidant systems (2), is at the basis of a wide range of diseases has become very popular both among scientists, with over 150,000 papers indexed in PubMed, as well as the lay public (3). A number of studies and meta-analyses of published trials have pointed out the lack of evidence to support the use of antioxidants in the prevention or treatment of several diseases, with some data suggesting a negative impact on disease [see, for instance, Ref. (4–6)]. We discuss elsewhere that, in the scientific literature, we often overestimate the evidence in favor of the oxidative stress theory of disease, often confusing association with causation, and overstate the potential usefulness of antioxidants (3). Despite the lack of evidence, there is a huge market of antioxidant supplements taken, without medical advice, in the hope to prevent or cure disease (3, 7).

The purpose of this study is to analyze the information on antioxidants available on the Internet to gather a picture on the public understanding on this topic. For this purpose, we used Google, the search engine used by over 65% of Internet users (8), to collect a significant sample of websites, and we downloaded the first 200 URLs in the search engine result page (SERP). These were analyzed in terms of the disease process they mention, whether they described antioxidants as contained in fruit or vegetables or as supplements. We also analyzed whether they had a positive or negative stance about antioxidants. Finally, we classified the websites as per their typology (e.g., governmental, commercial, no-profit, news, and professional websites).

The analysis of a relatively large sample provided a snapshot of the public understanding of antioxidants in health and evidentiates the focus of commercial interests and potential misinformation. It also showed that the usefulness of antioxidant supplements is controversial, with some sources of information stressing risks and others benefits.

We analyzed them for standard measures of trustworthiness in health information quality (HIQ). There are some established criteria used in the literature for measuring HIQ and most of them are, in fact, indicators of trustworthiness, the most basic dimension of HIQ. For instance, the most used criteria are the JAMA score, described the Journal of the American Medical Association (9). This analyses on the presence of four elements: the names of the authors, the date of writing or update, references to sources, and indication of owners of the website. A different measure of HIQ is the presence of the Health-on-the-Net certification (HONCode) (10). This certification is provided by an independent organization and examines transparency criteria (including those of the JAMA score) but also takes into consideration ethical principles, such as whether the website intends to replace, rather than complement, the doctor. Another instrument, DISCERN, is targeted at websites about drugs and considers other criteria linked to transparency or ethics, such as whether they describe both benefits and risks of a drug, the overall effect on quality of life, and the comparison with other treatment choices (11).

These instruments, however, do not analyze the content of the website and whether the information provided is scientifically correct. An assessment of the scientific correctness (“accuracy”) of the information provided against current guidelines and medical knowledge about pathophysiology, diagnosis, and treatment requires evaluation by a panel of expert (12).

We proposed, as a proxy to scientific correctness, to analyze whether a website points to a treatment that has been approved by a regulatory agency based on strong evidence-based medicine (13, 14). However, we should bear in mind that, so far, no antioxidants are approved for any indication. The only antioxidant approved for clinical use is edaravone, for stroke and amyotrophic lateral sclerosis, and only in Japan (15).

Finally, we also analyzed the way Google ranks these websites, performing a sub-analysis of the top 10 websites in the SERP. In fact, it is known that users are more likely to only look at the first websites shown (16), and there is a common belief that search engines such as Google will give higher visibility to commercial websites.

## Materials and Methods

### Data Collection

We searched the term “antioxidants” on 10 December 2015 in http://Google.com using the browser Firefox (Mozilla, Mountain View, CA, USA) after logging out from any Google account, clearing caches and browsing history to avoid the results to be influenced by previous searches [the so-called “bubble effect,” although this is probably not a major issue in health-related searches (17)]. The first 200 URL returned in the SERP were transferred to a spreadsheet using the Firefox extension SEOquake (SEMrush, Trevose, PA, USA). Each URL was then visited independently and assessed. Inclusion and exclusion criteria for initial assessment in the Excel spreadsheet were as follows: the webpage must be in the English language, must be freely accessible c (i.e., paywalls and log in requirements excluded the webpage from the assessment), duplicate URLs were excluded from further analysis. We also excluded those that mentioned the word “antioxidant” but outside a disease context. As a result, 144 webpages were included in the analysis.

### Classification of Websites

Initial assessment of each URL involved categorization of the following:

(1) Diseases or biological process (e.g., aging, immunity) mentioned in the webpage from a list of the most commonly mentioned diseases/biological processes in the SERP: cancer, diabetes, cerebrovascular and cardiovascular disease (CVD), neurological disease (such as Alzheimer’s and Parkinson’s diseases), eye disease, arthritis, immune functions, and aging.
(2) The context of antioxidant discussion on the webpage, i.e., context of diseases/body systems where antioxidants are mentioned.
(3) The viewpoint of the webpage, i.e., pro, against, or neutral regarding antioxidants from food sources and/or supplements. Examples of statements showing a negative stance are given in Table 1.
(4) HON code certification present or not. The HON code certification is detected by the presence of a symbol, the HONCode seal, which can be clicked to check with the health-on the-net website to check the currency of the certification.
(5) The JAMA score of the webpage. To calculate the JAMA score, we analyzed each webpage for the presence of following information in the page: author name, date of publication, or update; disclosure of ownership of the website; and attribution (presence of references or sources for the information provided). Each criterion present would score 1; therefore; the JAMA score is in the range 0–4.

**Table 1:** **Examples of negative statements on antioxidants**.

Text string	Website (archived URL in parenthesis)
High-dose supplements of antioxidants may be linked to health risks in some cases. For example, high doses of beta-carotene may increase the risk of lung cancer in smokers	https://www.nlm.nih.gov/medlineplus/antioxidants.html (http://www.webcitation.org/6jdOjgv98)

More recently, a 2011 trial involving more than 35,500 men over 50 found that large doses of vitamin E increased the risk of prostate cancer by 17 percent	http://www.scientificamerican.com/article/antioxidants-may-make-cancer-worse/ (http://www.webcitation.org/6jdQoyIDk)

For people with an increased risk of cancer, this means that taking nutritional supplements containing antioxidants may unintentionally speed up the progression of a small tumor or premalignant lesion, neither of which is possible to detect	https://www.rt.com/usa/318710-antioxidants-metastasize-melanoma-cancer/ (http://www.webcitation.org/6jdTeL0dj)

In 2007, a combined analysis of 68 randomized trials of any antioxidant supplements showed a statistically significant 5% increase in risk of death in the groups taking the supplements compared to the groups taking placebo pills	http://www.rawstory.com/2015/10/antioxidants-can-protect-our-cells-but-antioxidant-supplements-are-generally-harmful/ (http://www.webcitation.org/6jdTm38FB)

Antioxidants are found in a variety of foods and dietary supplements and are frequently used with the goal of preventing cancer, but mounting evidence suggests that they may not be as beneficial as once thought. Clinical studies have shown mixed or no benefits, and other works demonstrated that antioxidants may accelerate the progression of lung cancer	http://stm.sciencemag.org/content/7/308/308re8

Rds. Tuveson and Chandel propose that taking antioxidant pills or eating vast quantities of foods rich in antioxidants may be failing to show a beneficial effect against cancer because they do not act at the critical site in cells where tumor-promoting ROS are produced—at cellular energy factories called mitochondria	http://www.cshl.edu/news-a-features/scientists-propose-how-antioxidants-can-accelerate-cancers-and-why-they-dont-protect-against-them.html (http://www.webcitation.org/6jevz7lIp)

A stipulation for scoring the webpages was that any information being scored must be present within three clicks from the webpage (however, links to external URLs were not allowed). The reason for the three-click rule (18) is to have some form of sensible accessibility to information, especially as information quality in this study is being assessed from the perspective of the public (19).

### Classification of Typology of Websites

We also classified websites as per their typology (government, professional, news, non-profit, health portal/blog, commercial, scientific journal, other) as previously described (13, 14); examples of these typologies in the present search are given in Table 2. Of note, we defined commercial websites of those who were selling a product or a book through the website; presence of advertising was not a criterion for considering a website as commercial.

**Table 2:** **Examples of websites typologies**.

Typology	Examples
Government	http://www.cancer.gov/
	http://www.internationaloliveoil.org/

Professional	http://www.aoa.org/
	http://my.clevelandclinic.org/

News	http://scientificamerican.com/
	https://www.washingtonpost.com
	http://www.besthealthmag.ca

Non-profit	https://en.wikipedia.org/wiki/Antioxidant
	http://www.antioxidants.org/

Health portal	http://www.webmd.com
	http://www.healthline.com

Commercial	http://www.healthchecksystems.com/
	http://articles.mercola.com/

Scientific journal	http://www.sciencedirect.com/science/article/pii/S002364380500188X
	http://www.nejm.org/doi/full/10.1056/NEJMcibr1405701

### Statistical Analysis

Statistical analysis was performed using GraphPad Prism for MacOS, version 7.

## Results

### Validation of the Classification of Websites

While the classifications for disease or antioxidant were easily identified by a string of text or a word, we noted that the classification by typology of websites was more subjective. Therefore, the classification of the typology of websites was performed independently by two authors and disagreements were resolved by discussion with a third author. The inter-rater variability in the classification of the typology of websites was analyzed using GraphPad. The observed agreements were 106 of 144 [73.6% of the observations resulting in a weighted Kappa = 0.671, 95% confidence interval (0.584, 0.759)], an agreement is considered “good strength” (20). However, the percentage of agreement varied with the typology of websites as follows: scientific papers, 100%; professional, 91%; commercial, 78%; news, 77%; government, 67%; no-profit, 63%; health portals, 60%; other, 44%. Due to the low inter-rater agreement, and because “others” included websites that were difficult to classify rather than having common features, these were not included in many of the analyses.

### Distribution of Websites by Typology or Disease/Biological Process

**Figure 1:**
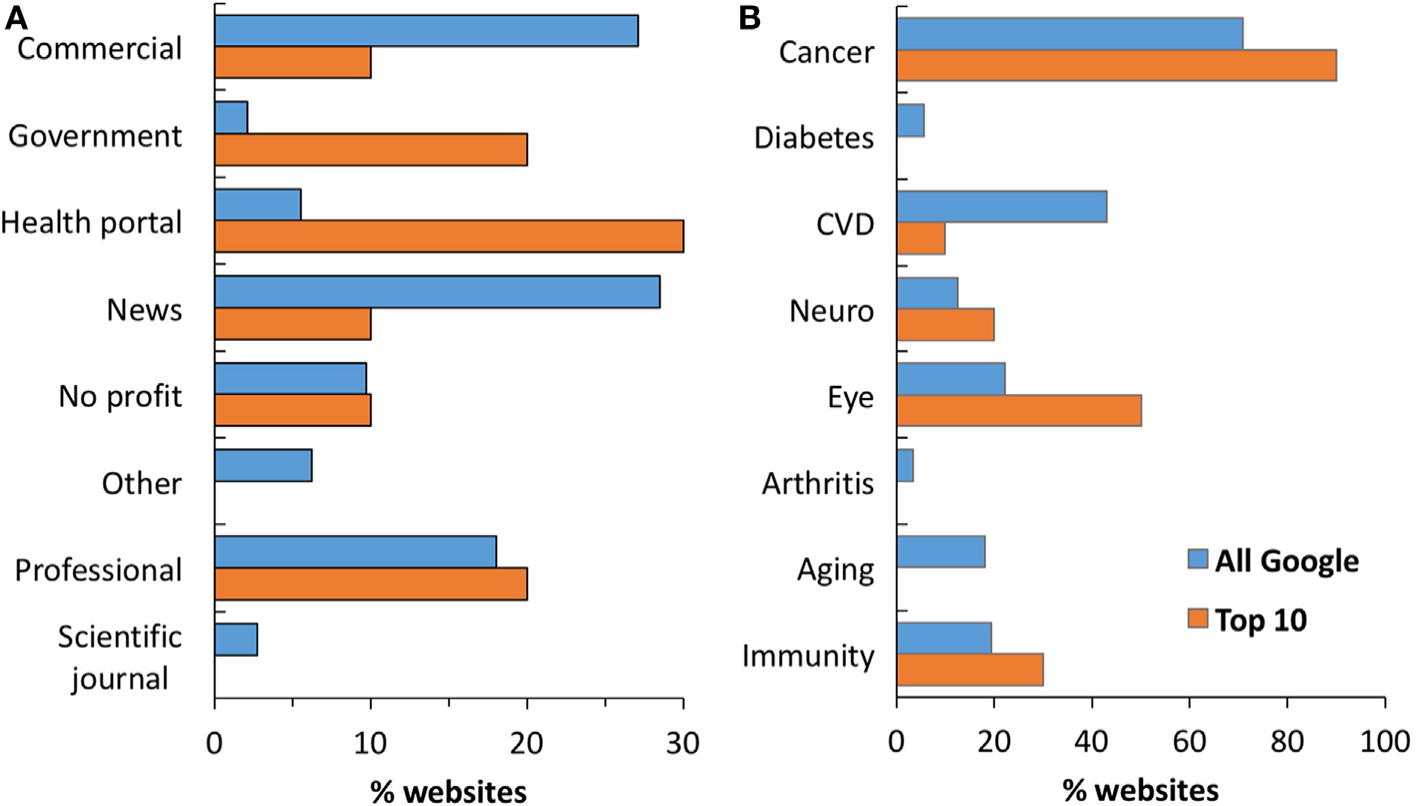
**Distribution of websites by typology (A) and biological process (B)**. Data show the percentage of websites in the top 10 results (orange, *n* = 10) and the total website in the search (blue, *n* = 144). In panel **(B)**, percentages do not add up to 100 as websites typically mention more than one disease/process.

Figure 1A shows the breakdown on the SERP in terms of class of website. Of the 144 websites analyzed, the most frequent typologies were (blue bars) news (28%) and commercial websites (27%), followed by professional websites (19%). However, in the top 10 web pages returned by Google (orange bars), news and commercial websites were less frequent (both 10%), with health portals (30%), government (20%), and professional (20%) websites being more represented. In fact, of the three government websites, two were in the top 10 (20%), compared to just one in websites 11–144 (0.8%). This was a statistically significant overrepresentation of government websites in the top 10 (*P* < 0.0001 by two-tailed Chi-square test). In fact, if the government websites were randomly distributed, their frequency in the top 10 and the bottom 134 should be the same of the whole search (0.8%). Likewise, an overrepresentation of health portals was also observed (out of 8, 3 were in the top 10 Vs. 5 in websites 11–144; *P* = 0.0005). An opposite trend was observed for commercial websites that were three times less frequent in the top 10. Although this difference was not significant, this trend is consistent with what we noted in previous studies on different health queries (13, 14).

Figure 1B shows the distribution of disease/processes mentioned by websites. It is important to note that, unlike the typology of a website, each website can mention more than one disease, thus percentages can add up to more than 100. Cancer was mentioned most frequently (in 102 websites, 70%) followed by cardiovascular and cerebrovascular disease (43%). Aging, immunity and eye diseases, were all mentioned by about 20% of the websites. There was no significant difference in this pattern between the whole SERP (blue bars) or the top 10 websites (orange bars). A list of the top 10 websites is provided in Table 3.

**Table 3:** **Top 10 websites in the Google search engine result page**.

Original URL	Archived website
https://www.nlm.nih.gov/medlineplus/antioxidants.html	http://www.webcitation.org/6dXNMmh8Z
https://en.wikipedia.org/wiki/Antioxidant	http://www.webcitation.org/6dXNQaOFt
http://www.hsph.harvard.edu/nutritionsource/antioxidants/	http://www.webcitation.org/6dXNT3qw3
http://www.cancer.gov/about-cancer/causes-prevention/risk/diet/antioxidants-fact-sheet	http://www.webcitation.org/6dXNWd7yR
http://www.webmd.com/food-recipes/antioxidants-your-immune-system-super-foods-optimal-health	http://www.webcitation.org/6dXNZsztz
http://www.webmd.com/food-recipes/antioxidants-topic-overview	http://www.webcitation.org/6dXNdV0ZF
http://familydoctor.org/familydoctor/en/prevention-wellness/food-nutrition/nutrients/antioxidants-what-you-need-to-know.html	http://www.webcitation.org/6dXNjj9aN
http://articles.mercola.com/antioxidants.aspx	http://www.webcitation.org/6dYP2MqVZ
http://www.theatlantic.com/health/archive/2011/10/antioxidants-explained-why-these-compounds-are-so-important/247311/	http://www.webcitation.org/6dYP7lJa0
http://www.medicinenet.com/script/main/art.asp?articlekey=11291	http://www.webcitation.org/6dYPI8u8d

### Viewpoint toward Antioxidants Normally Present in Foods Vs. Supplements

We analyzed the text in terms of which source of antioxidant was mentioned. Of the 144 websites, 106 mentioned antioxidants contained in food and 105 those contained in supplements, 72 websites mentioned both food and supplements. The two antioxidants mentioned more frequently were vitamin E (99 websites) and vitamin C (84 websites).

**Figure 2:**
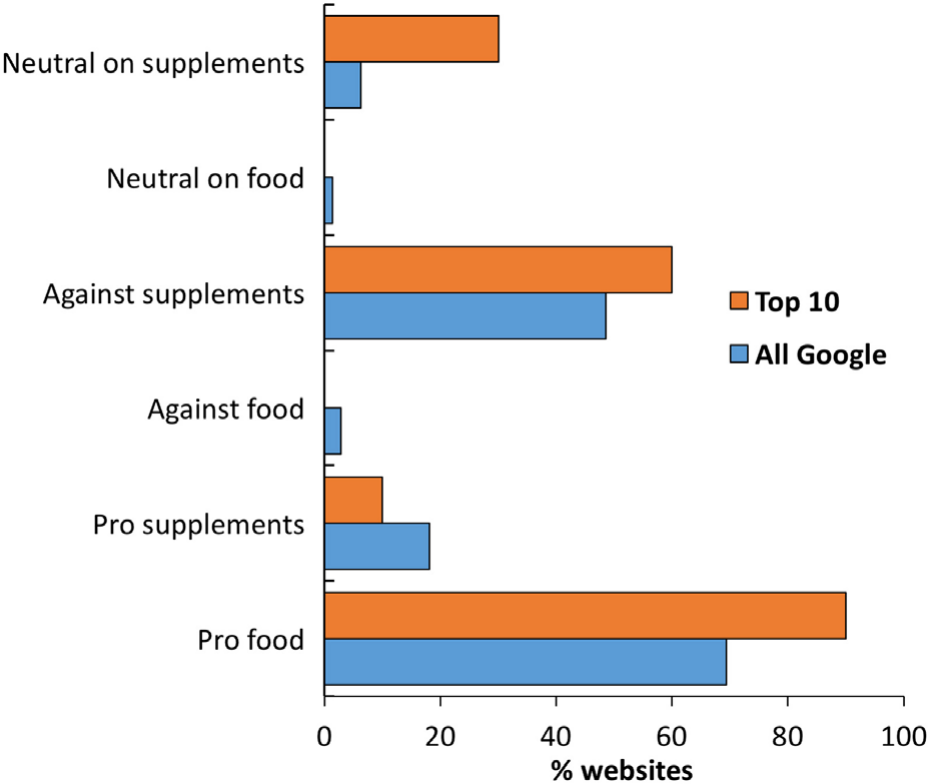
**Analysis of websites by stance on antioxidants in food or as supplements**. Data show the percentage of websites in the top 10 results (orange, *n* = 10) and the total website in the search (blue, *n* = 144).

We read the text of each website to understand whether they expressed a positive or negative view of antioxidants, making a distinction between antioxidants present in food or as supplements. As shown in Figure 2 (blue bars), there was a large consensus for a positive view on antioxidants present in food (70% of webpages) and a negative view of antioxidant supplements [with much less in favor on antioxidant supplements (18%)]. This pattern was very similar in the top 10 websites returned by Google (orange bars).

We, therefore, performed a sub-analysis of the viewpoint by typology of websites or by disease/biological process mentioned of websites. The results are shown in Tables 4 and 5 as the percentage of expected website with a specific viewpoint (as found in the whole SERP of 144 websites) and the percentage observed.

**Table 4:** **View on antioxidants in different types of websites**.

	For food	For supplements	Against food	Against supplements	Neutral for food	Neutral for supplements	No in websites in typology
Government	67% (2)	0	0	67% (2)	0	0	3
Professional	75% (21)*	7% (2)	7% (2)	64% (18)*	0	7% (2)	26
News	66% (27)	0	2% (1)	59% (24)	5% (2)	0	41
Commercial	59% (23)	44% (17)**	0	18% (7)**	0	10% (4)	39
Non-profit	86% (12)	0	0	57% (8)	0	14% (2)	14
Health portal	88% (7)	13% (1)	0	75% (6)	0	13% (1)	8
Other	78% (7)	33% (3)	11% (1)	55% (5)			9
Scientific journal	25% (1)						4
Expected in total search	48% (69)	13% (18)	2% (3)	34% (49)	1% (1)	4% (6)	144

Data indicate that the percentage of observed websites in that category (for food, for supplements, against supplements) is calculated as no. website in each cell/no. websites in the rightmost column. Table is color coded: cells in red indicate whether a typology is overrepresented (higher than the expected percentage in the respective category/stance, indicated in the bottom row), in blue if it is underrepresented. Comparison was performed vertically, i.e., number of websites with that stance [e.g., government for food were 2 out of 3 (67%) and were compared with the number of websites “for food” in the total search, 69 out of 144 (48%) using a Fisher’s exact test]. *P*-value was adjusted with the Bonferroni’s correction for a total of *n* = 8 vertical comparisons (*P < 0.05; **P < 0.005).

**Table 5:** **View on antioxidants by disease mentioned in websites**.

	For food	For supplements	Against food	Against supplements	Neutral for food	Neutral for supplements	No websites in disease category
Cancer	75% (77)	6% (6)	2% (2)	60% (58)	2% (2)	7% (7)	102
Diabetes	100% (8)	0	0	0	0	0	8
Cardiovascular disease	84% (52)	19% (12)	0	34% (21)	0	7% (4)	62
Neuro	89% (16)	17% (3)	0	33% (6)	0	17% (3)	18
Eye	84% (27)	31% (10)	0	25% (8)	0	16% (5)	32
Arthritis	80% (4)	20% (1)	0	60% (3)	0	0	5
Aging	62% (16)	50% (13)*	8% (2)	23% (6)	0	0	26
Immunity	68% (19)	46% (13)*	0	21% (6)	0	14% (4)	28
Expected in total search	69% (100)	18% (26)	3% (4)	49% (70)	1% (2)	6% (9)	144

Data indicate that the percentage of observed websites in that category (for food, for supplements, against supplements) is calculated as no. website in each cell/no. websites in the rightmost column. Table is color coded: cells in red indicate whether a typology is overrepresented (higher than the expected percentage in the respective category/stance, indicated in the bottom row). Comparison was performed vertically, i.e., number of websites with that stance [e.g., government for food were 2 out of 3 (67%) and were compared with the number of websites “for food” in the total search, 69 out of 144 (48%) using a Fisher’s exact test]. *P*-value was adjusted with the Bonferroni’s correction for a total of *n* = 8 vertical comparisons (*P < 0.05).

Table 4 shows a breakdown by typology of websites. It is clear that commercial websites have a higher “pro-supplement” stance than expected. This should be interpreted as an unbalance, rather than a polarization, as it is paralleled by an underrepresentation of “against supplements” views. Likewise, Table 5 shows a breakdown by disease or biological process. A pro-supplement stance was significantly higher for aging or immunity.

Thus, cancer is more likely to be associated with a viewpoint negative about antioxidant supplements, while, on the contrary, aging and immunity are more likely associated with a positive view of antioxidant supplements. Additionally, while a pro-antioxidants-in-food stance does not show any marked difference among the types of websites, a pro-supplement stance is highly represented in commercial websites. In the entire SRTP, of the 102 websites mentioning cancer, 50 had a favorable view of antioxidants, 46 had a negative view (mentioning the potential risk of increasing cancer), and 6 represented both views. The stance towards antioxidants in cancer (positive or negative) was analyzed in the main typologies of websites. The frequency of commercial websites in favor of antioxidants was over fivefold that in websites against antioxidants (17/50, 34%, Vs. 3/46, 6.5%; *P* = 0.0009 by two-tailed Chi-square test). On the contrary, news websites represented a significantly higher proportion of negative views than expected (positive, 10/50, 20%, Vs. negative, 22/46, 48%; *P* = 0. 0.0039).

Because commercial websites had the highest frequency of positive views of antioxidants, we analyzed their proportion in websites as per the disease areas mentioned and see the higher proportion of a pro-supplement stance in some areas could be due to a higher proportion of commercial websites. Also, in view of the previous observation, we split the websites mentioning cancer into those with a positive view or with a negative view of antioxidants.

**Figure 3:**
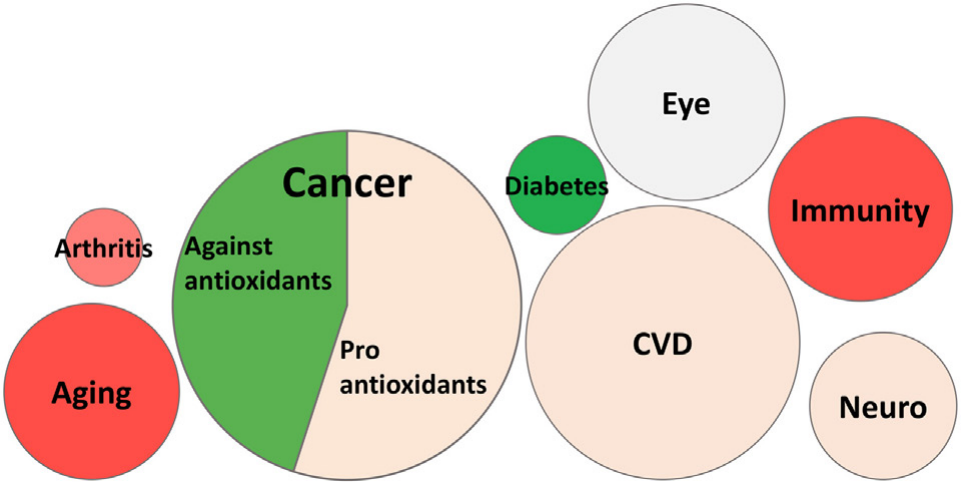
**Percentage of commercial websites by different diseases/processes**. The area of the bubbles is proportional to the number of websites mentioning each process/disease. Data are color coded to indicate whether they differ from expected percentage of commercial websites (27% in the entire sample of 144 websites): red >27%; green <27%. In the case of cancer, we separated websites with a positive or negative stance on antioxidants. Percentage of commercial websites is shown for each process/disease.

The results in Figure 3 indicate that websites on diabetes (*n* = 8) and those with a negative view on antioxidants in cancer (*n* = 46) have a much lower representation of commercial websites, while those mentioning the two broad biological processes of aging or immunity had the highest proportion of commercial websites.

Finally, we have not analyzed the four papers in scientific journals that were present in the SERP. In previous studies, we did not find scientific studies making it to the 200-long Google SERP. Although the four webpages which did not rank high (they were 48th, 62nd, 103rd, and 132nd), it is interesting that one of them was a commentary in the New England Journal of Medicine highlighting the potential negative effects of antioxidants in cancer (21), and two were webpages were a science advisory from the American Heart Association of 2004 about the lack of benefits of antioxidants in CVD (22). Another webpage, for the Proceedings of the National Academy of Sciences 2009 experimental study reported that antioxidant administration prevents health-promoting effects of exercise (23).

### Analysis of the JAMA Score and HONCode Certification of Websites

The JAMA score of the overall search was median 2, IQR [1, 3] with 10 websites (6.9%) displaying the HONCode certification. The median JAMA score in the HONCode certified websites was higher than in the non-certified websites (median 2.5, IQR [2, 4] Vs. median 2.0, IQR [1, 3]) although the difference was just below the statistical significance (*P* = 0.0525 by two-tailed Mann–Whitney’s test).

**Figure 4:**
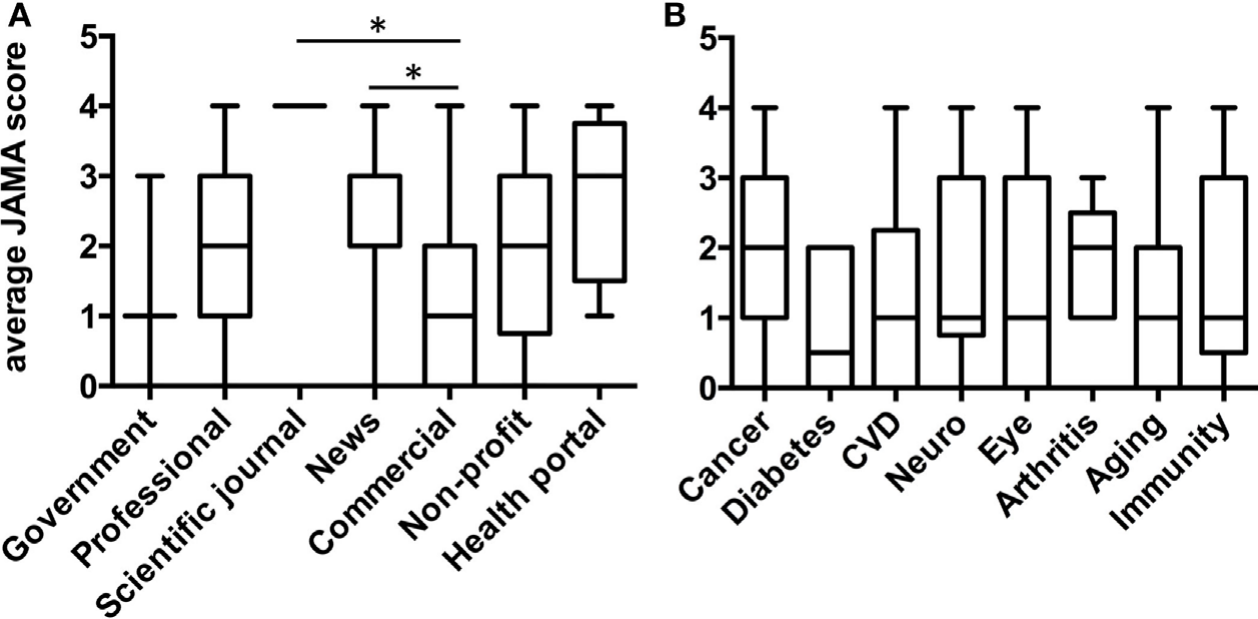
**JAMA score by typology of website (A) or disease/process mentioned (B)**. Data are reported as median and interquartile range. Significantly different (*P = 0.005; two-tailed Kruskal–Wallis test, followed by the Dunn’s test).

Figure 4A shows the median JAMA score in the different classes of websites. The highest value was observed in news (*n* = 41) websites and health portals (*n* = 8), the lowest in commercial (*n* = 39) and government websites (*n* = 3). Multiple comparisons showed that the JAMA score of news websites (median 2, IQR [2, 3]) was significantly higher than that of commercial websites (median 1, IQR [0, 2]). In an analysis by disease or process, shown in Figure 4B, the highest JAMA score was observed in websites mentioning cancer or arthritis (both medians = 2).

Finally, of the 102 websites containing information on cancer, those with a positive stance of antioxidants (*n* = 50) had a median JAMA score of 1.5, IQR [1, 3], while those with negative stance (*n* = 46) had a median JAMA score of 3, IQR [2, 3], a difference that was statistically significant (*P* = 0.0005 by two-tailed Mann–Whitney’s test).

### Google Ranking

The JAMA score of the top 10 websites presented by Google was slightly higher, but not significantly, from that of the remaining 134 websites (median 2.5, IQR [1, 4] Vs. 2, IQR [1, 3]) while the number of HONCode-certified websites was significantly higher in the top 10 websites than in the remaining websites (4/10 Vs. 6/134; *P* < 0.0001 by two-tailed Chi-square test).

In the top 10 websites, the JAMA score of the four HONCode-certified websites was significantly higher than that of the 6 non-certified websites (median 4, IQR [4, 4] Vs. median 2, IQR [1, 2.25]; *P* = 0.0048 by two-tailed Mann–Whitney’s test).

## Discussion

The present study gives a comprehensive picture of the information available on the web on antioxidants, focusing on their role in disease conditions or broad biological processes such as aging and immunity, and on the impact of commercial websites and the information they provide.

The descriptive analysis of the webpages shows that commercial websites and news websites are the two largest class of websites. This may raise concern on the quality of information on antioxidants as that most websites (over half of the entire list) are commercial websites (many of which commercialize antioxidant supplements) and news websites [presumably containing information largely based press releases, often of poor quality (24, 25) or themselves written by companies selling supplements (26)].

However, while Google returns several commercial websites, this probably reflects what is available on the Internet. On the other hand, commercial websites are not ranked well by Google. On the contrary, the few government websites and health portal are ranked highly, being the two largest classes in the top 10 website in the Google SERP. This, along with the low representation of commercial websites in the top 10 returned by Google, is something we noted consistently in previous studies (13, 14) and might reflect the fact that they have better IQ features. While the algorithm used by Google is not public, and various IQ criteria might account for the lower ranking of commercial websites (in-links, out-links, sharing by social networks, etc.), it is notable that the ranking has the same trend as the JAMA score or the HONCode certification, suggesting that intrinsic dimensions of IQ might be important in the ranking. It is unfortunate that most studies of HIQ that analyzed typologies of websites and various HIQ criteria do not provide the original SERP with the Google ranking as a supplementary file to verify whether our observation was reproduced in other studies.

To summarize, the main findings from the analysis of the typology of websites and their HIQ trustworthiness indicators were (1) commercial and news websites are the most frequent on a search on antioxidants; (2) the ranking by Google promotes government websites and health portals while penalizing commercial websites; and (3) Google ranks higher websites with the HONCode certification and a higher JAMA score.

When the content of the webpages was analyzed in terms of topics discussed and their stance about antioxidants, the fact that commercial websites would try to promote their products and had a positive stance on antioxidants was not surprising.

We were surprised to find that news websites reported, prevalently, news that had a negative view of antioxidants. Reading through those websites, we noted that many of the websites mentioning a negative effect of antioxidants in cancer were referring to two papers published in 2015 reporting that antioxidant supplements can increase cancer progression in mice (27, 28) or counteract the health-promoting effects of exercise (23). It is difficult to say whether this was accidental, because of the high impact of those two scientific papers. It may be that those studies were considered more newsworthy because newspapers and news websites are more attracted by “bad news,” as discussed elsewhere (13, 29). It is curious that the four webpages from academic journals that “made it” into the SERP were all of studies in top journals that reported a lack of benefits or a risk associated with antioxidants, as discussed above.

The different proportion of a pro- Vs. anti-supplement view in other diseases/biological process is intriguing. This information should be analyzed in the context of the “popularity” of that condition (in terms of number of webpages mentioning it) as well as the strength of the evidence for a role oxidative stress, and by consequence of the effect of antioxidants, for the specific condition (3). We discussed above the possible reasons for a strong representation of a negative view on antioxidants in cancer. At the other extreme, there is a high proportion of pro-supplement stance among websites describing immunity or aging. This may well be because there are more basic studies on oxidative stress and antioxidants on general biological processes than on specific diseases. It is difficult to compare results from a PubMed search (http://www.ncbi.nlm.nih.gov/pubmed) with one in Google because terms not always match. However, searching on “antioxidants” and some of these diseases/processes (using wild cards) on 25/08/2016 gave a different distribution. While cancer was still first (43,000 papers), diabetes was second (15,000) followed by neurological diseases (19,000), aging (14,000), and immunity (12,000). Of course, the number of studies published does not equal the number of studies showing a positive effect of antioxidants in a disease model. However, we can safely assume that the publication bias is such that, in the preclinical scientific literature, mainly positive results with antioxidants will be published (30, 31). Thus, the number of scientific publications on the topic does not explain the higher proportion of pro-supplement websites mentioning aging or immunity shown in Table 5. It is possible that this is due to the high percentage of commercial websites, as shown in Figure 3.

There may be many reasons why marketing of antioxidant supplements and alternative medicine targets these two broad areas. It is possible that consuming antioxidant to “boost immunity” or “improve aging” might ensure usage over a longer period and in healthy people. It may also be that FDA regulations are more lenient for supplements, not requiring the high level of evidence needed for drug approval, if there are no claims that the supplement could cure a disease (32).

In conclusion, from the content analysis, we found that (1) cancer and vascular disease where those mentioned more frequently; (2) while most webpages had a positive stance on antioxidants contained on foods, a negative view was prevalent for antioxidants as supplements, and this was particularly evident for cancer; (3) a negative view on antioxidants was prevalent in news webpages while a pro-antioxidant view, including supplements, was prevailed in commercial webpages; and (4) of the disease/processes, aging and immunity had the highest proportion of commercial websites. Different diseases are differently associated with the idea that antioxidant supplements may be good or bad for your health and this depend on the presence of commercial websites that, for obvious reasons, have a pro-supplement stance. Processes like aging and immunity, rather than specific diseases, are the ones with a more pronounced pro-supplement and commercial component.

The study has some limitations and care should be taken before extrapolating them to other areas. Obviously, the websites returned depend on the search terms used and the date of the search, so we must be careful in generalizing. On the other hand, having analyzed 200 websites returned provides a reasonable sample of the existing information on the web, irrespectively of how these are ranked by Google. News websites are time-sensitive as most of them will just reflect press releases.

Location of the search may also be an issue determining which websites are returned, although in our case, we forced the browser to use http://Google.com rather than the default local version (http://google.co.uk), although the IP address from where the search will always be associated with a geographical location. Another issue is that of whether previous history affects subsequent searches. This should not apply to us as we deleted the cookies and browsing history before performing the search. Also, the fact that, in Google, previous search history can significantly modify the results of subsequent searches is a widespread belief, largely because of the popular book “The Filter Bubble” (33), but there is little evidence of it and a study on Google searches with virtual agents performing 64,000 health-related queries has shown no evidence of results present in the SERP being influenced by the previous history (17).

Another major limitation was that some typologies (health portals, no-profit, and “other”) are ill-defined and their classification subjective, resulting in a low inter-rater agreement (<75%). Therefore, any conclusions regarding these classes of websites should be taken with great caution. Also, we did not consider Google Ads. Although these are clearly labeled as advertisements, they may still be read as they are shown before the SERP. Specific studies should be done to assess HIQ in websites labeled as advertisements.

Finally, we only assessed some aspects of these websites, while IQ has several dimensions (34) that might be important in determining whether a layperson will actually read a website in the SERP. These include, for instance, loading speed (8 s being considered an upper limit by most, or readability). It will be important to evaluate the various weight of these different components and whether the criteria for HIQ are different for different readers such as patients, doctors, or other health professionals, and extend the analysis to social media.

## Author Contributions

RA, DG, and PG performed research and wrote the manuscript.

## Conflict of Interest Statement

The authors declare that the research was conducted in the absence of any commercial or financial relationships that could be construed as a potential conflict of interest.
